# Low prevalence of *Contracaecum* third-stage larvae parasitizing Sea of Galilee fisheries: 1-year survey after 57 years of no information

**DOI:** 10.1016/j.fawpar.2023.e00204

**Published:** 2023-07-08

**Authors:** Nadav Davidovich, Perla Tedesco, Monica Caffara, Valentina Luci, Alessia Cantori, Danny Morick, Maria Letizia Fioravanti, Andrea Gustinelli

**Affiliations:** a Israeli Veterinary Services, Bet Dagan 5025001, Israel; b Department of Veterinary Medical Sciences, Alma Mater Studiorum – University of Bologna, 40064 Ozzano Emilia, BO, Italy; c Morris Kahn Marine Research Station, University of Haifa, Haifa 3498838, Israel; d Hong Kong Branch of Southern Marine Science and Engineering Guangdong Laboratory, Guangzhou, Hong Kong, China

**Keywords:** *Contracaecum* larva, Zoonosis, Fishery, Sea of Galilee, Israel

## Abstract

Freshwater and marine ecosystems are a suitable habitat for parasitic nematodes of the genus *Contracaecum* (family: Anisakidae) to complete their complex life cycle. Several fish species of the Sea of Galilee (Lake Kinneret) were reported in 1964 as second intermediate/paratenic hosts of *Contracaecum* spp. larvae. The lack of taxonomically relevant morphological features of these larvae hindered their proper identification. Here we report the results of a 1-year survey conducted in 2021, 57 years after the first (and only) such survey. We analyzed 352 specimens from 10 fish species (native and non-native) of the Sea of Galilee (Israel) ichthyofauna. We compared our results with those of the first parasitological survey conducted by Paperna in 1964; the overall prevalence of nematodes referable to *Contracaecum* larvae was 16.8% and 0.85% in 1964 and in 2021, respectively. Different from the first survey that identified *Contracaecum* larvae morphologically, we used both morphological and molecular tools. Two wild native cyprinids—Jordan himri (*Carasobarbus canis*) and Jordan barbel (*Luciobarbus longiceps*)—were infected (a single specimen each) with *Contracaecum quadripapillatum* larvae in their abdominal cavity. A single specimen of blue tilapia (*Oreochromis aureus*) was infected with two larvae of *Contracaecum multipapillatum* E, localized in the pericardial cavity. The findings of our study, which is part of a large project focused on *Contracaecum* spp. infecting both piscivorous birds and fish collected in Israel, advance our knowledge about the distribution and host range of this potentially zoonotic parasite in fishery products of the Sea of Galilee.

## Highlights

• 352 specimens belonging to ten fish species from the Sea of Galilee analyzed
• Dramatic reduction in the prevalence of *Contracaecum* spp. as compared to 1964
• First molecular data of *Contracaecum* larvae in Sea of Galilee fisheries
• First record of *C. quadripapillatum* in wild native cyprinids
• First confirmation of *C. multipapillatum* E larvae in wild blue tilapia

## 1 Introduction

Parasitic nematodes of the family Anisakidae naturally parasitize poikilothermic organisms (fish and aquatic invertebrates) and homeothermic organisms (marine mammals and fish-eating birds) as intermediate/paratenic hosts and definitive hosts, respectively, with humans becoming accidental hosts when eating raw fish infected with the third-stage larvae (L3) (Nagasawa, 2012). The Anisakidae family includes, among others, three genera such as *Anisakis*, *Pseudoterranova* and *Contracaecum* of zoonotic/potentially zoonotic importance (Buchmann and Mehrdana, 2016). Larval nematodes belonging to this family are widespread in wild and farmed fish populations worldwide (Shamsi, 2019). The genus *Contracaecum* consists of over 60 species which mature in fish-eating birds, mainly of the families Pelecanidae (Landsberg, 1989; Mattiucci et al., 2010), Phalacrocoracidae and Ardeidae (Shamsi, 2019) but also marine mammals and penguins (Shamsi et al., 2009; Garbin et al., 2019).

The Sea of Galilee (Lake Kinneret or Lake Tiberias) is a monomictic lake, considered to be the largest (21 km long and 13 km wide) surface freshwater reservoir in the Middle East; it is located in the Syrian–African Rift Valley in the northeastern part of Israel (Berman, 1998). The ichthyofauna of the Sea of Galilee includes 19 native and 8 exotic fish species (Goren and Ortal, 1999; Ostrovsky et al., 2014). The native fish species are dominated by members of the families Cyprinidae and Cichlidae (Goren and Ortal, 1999; Werner and Mokady, 2004). Of the eight exotic fish species in the lake, only two, common carp (*Cyprinus carpio*) and mosquitofish (*Gambusia affinis*), have established viable populations (Golani et al., 2019).

According to the Ministry of Agriculture and Rural Development, of the 175,000 tons of fish supplied to the Israeli market in 2018, less than 1% originated from local fisheries, including the Sea of Galilee and the Mediterranean Sea (Bar-Nahum, 2019). Several studies have described the biology and ecology of fish populations in the Sea of Galilee (Ostrovsky et al., 2014), including biomanipulation based on food-web modeling (Ofir et al., 2017). Recently, Shapiro et al. (2022) published a study on ecosystem changes related to fish diet and trophic chains. In contrast, studies on parasites affecting the fish in this area are scarce, and most of them refer to digenetic trematodes (Farstey, 1986; Finkelman, 1988; Yekutiel, 1985). Dzikowski et al. (2003) reported the presence of metacercariae of the family Clinostomidae in cichlids, and the relationship between these parasites and declining water level in the Sea of Galilee. Other studies describing metacercariae of the family Clinostomidae in the Sea of Galilee were published by Caffara et al., in 2014 and in 2016. Most recently, Falk et al. (2020) described the presence of some generalist protozoans, myxozoans and digeneans affecting cichlids. To the best of our knowledge, none of the papers published to date, except for Paperna (1964), has addressed parasites of the family Anisakidae, with reference to the genus *Contracaecum*, in fish of the Sea of Galilee. Paperna (1964) examined seven fish species collected in this area, reporting the presence of unidentified *Contracaecum* larvae with an overall prevalence of 16.8%, and briefly describing the few morphological characteristics available for this developmental stage.

Herein, we describe the results of a 1-year fish survey carried out in the same site examined by Paperna (1964) in the Sea of Galilee, 57 years after the first and only morphological report on *Contracaecum*, using both morphological and molecular approaches.

## 2 Materials and methods

### 2.1 Fish sampling

In 2021, a parasitological survey was carried out on 352 fish, mainly of the families Cyprinidae and Cichlidae, collected from the Sea of Galilee (see Table 1 for details).

**Table 1:** Comparative data of *Contracaecum* spp. infecting different fish species from the Sea of Galilee in 1964 by Paperna and in 2021 in the present study.

Fish Family	Fish species	Year 1964 (Paperna, 1964)		Year 2021 (present study)	
		No. of analyzed fish	Prevalence	No. of fish infected / fish examined	Prevalence (MI/MA)
Cyprinidae	Jordan himri (*Carasobarbus canis*) (formerly *Barbus canis*)	12	0	1/63	1.6 % (1/0.01)
	Jordan barbel (*Luciobarbus longiceps*) (formerly *Barbus longiceps*)	5	20 %	1/87	1.1% (1/0.01)
	Levantine scraper (*Capoeta damascina*) (formerly *Varicorhinus damascinus*)	5	25 %	0/2	0
	Levantine minnow (*Garra nana*) (formerly *Tylognathus steinitziorum*)	7	14.3 %	nt	nt
	Common carp (*Cyprinus carpio*)	7	0	0/40	0
Leuciscidae	Kinneret bleak (*Mirogrex terraesanctae*) (formerly *Acanthobrama terraesanctae*)	19	16.7 %	0/14	0
Mugilidae	Flathead grey mullet (*Mugil cephalus*)	nt	nt	0/8	0
	Thinlip grey mullet (*Chelon ramada*) (formerly *Mugil capito*)	1	2.6 %	nt	nt
Cichlidae	Blue tilapia (*Oreochromis aureus*)	nt	nt	1/29	3.4 % (2/0.07)
	Mango tilapia (*Sarotherodon galilaeus*) (formerly *Tilapia galileae*)	12	nt	0/92	0
	Tvarnun simon (*Tristramella simonis*)	12	0	0/6	0
	Redbelly tilapia (*Coptodon zillii*) (formerly *Tilapia zillii*)	19	14.3 %	0/11	0
Clariidae	North African catfish (*Clarias gariepinus*) (formerly *Clarias lazera*)	4	25 %	nt	nt

nt = not tested

MI = Mean Intensity

MA = Mean Abundance

The fish were caught by professional anglers using mesopelagic gillnets and purse seines and were kept refrigerated (+4 °C) until examination. In the laboratory, fish were measured, weighed, sexed, and then subjected to visual inspection of the abdominal cavity, internal organs, and musculature for the presence of zoonotic parasites following the procedure for fish premarketing control (Israeli Veterinary Services, 2017). Moreover, each filet was cut into thin slices (5 mm) and carefully inspected using a white light transilluminator as described by Menconi et al. (2020). Nematodes were isolated with a dissecting needle, counted, and preserved in 70% ethanol for both morphological and molecular analyses. Mean intensity and mean abundance were calculated following Bush et al. (1997).

### 2.2 Morphological examination

The larvae were observed under a light microscope (Leica Microsystems, Wetzlar, Germany) to record total length and for molecular analysis, a small portion (about 5 mm) that was devoid of taxonomical features was cut from the central part of the larvae. Anterior and posterior portions of the body were clarified in Amman's lactophenol to measure the internal taxonomical structures by light microscope with the aid of a digital Nikon DS-Fi1 camera and image-acquisition software (Nikon Nis-Elements D3.0). Morphometric analysis was carried out following Anderson (2000), Berland (1961) and Saad et al. (2018). Measures are given in micrometers unless otherwise indicated.

After measuring their anterior and posterior portions, the four nematodes were processed for scanning electron microscopy (SEM): they were dehydrated through a graded ethanol series, subjected to critical point drying, sputter-coated with gold palladium, and observed using a Phenom XL G2 Desktop SEM (Thermo Fisher Scientific, Eindhoven, The Netherlands) operating at 5 kV.

### 2.3 Molecular identification

For molecular analysis, genomic DNA was extracted from the central pieces of all four larvae found in this study, using a PureLink® Genomic DNA Kit (Life Technologies, Carlsbad, CA, USA) following the manufacturer's instructions. Amplification of the internal transcribed spacer (ITS) rDNA region was performed with the primers NC5_f (5′-GTAGGTGAACCTGCGGAAGGATCATT-3′) and NC2_r (5′-TTAGTTTCTTCCTCCGCT-3′). A 10- μL aliquot of the PCR product was subjected to PCR-restriction fragment length polymorphism (RFLP) with the enzyme *Msp*I (Caffara et al., 2023).

For sequencing, the amplicons were excised from a gel, purified by Nucleo-Spin Gel and PCR Clean-up (Macherey-Nagel, Düren, Germany), and sequenced in an ABI 3730 DNA analyzer (StarSEQ, Mainz, Germany). The DNA trace files were assembled with Contig Express (VectorNTI Advance 11 software, Invitrogen, Carlsbad, CA, USA), and the consensus sequences of the ITS rDNA and *cox*2 mtDNA were compared with previously published data by BLAST tools (https://blast.ncbi.nlm.nih.gov/Blast.cgi).

The sequences generated in this study have been deposited in GenBank under accession numbers OQ690009–10 (*C. quadripapillatum*) and OQ690011–12 (*C. multipapillatum* E).

## 3 Results

Overall, three out of 352 (0.85%) fish examined showed non-encysted *Contracaecum* larvae: two larvae from one blue tilapia were in the pericardial cavity, whereas both Jordan himri and Jordan barbel specimens were infected in the abdominal cavity by only one *Contracaecum* larva each. All of the other examined fish species were negative (Table 1).

The RFLP produced two different restriction patterns: two larvae (from Jordan himri and Jordan barbel) showed bands of 360–600 bp corresponding to *C. quadripapillatum*, whereas the two other specimens (from blue tilapia) were undigested and tentatively identified as *C. multipapillatum* E. The ITS rDNA sequences obtained compared by BLAST with the sequences available in GenBank, showed 99.9% identity with *C. quadripapillatum* and 99.8–100% with *C. multipapillatum* E, confirming the RFLP identification.

**Fig. 1:**
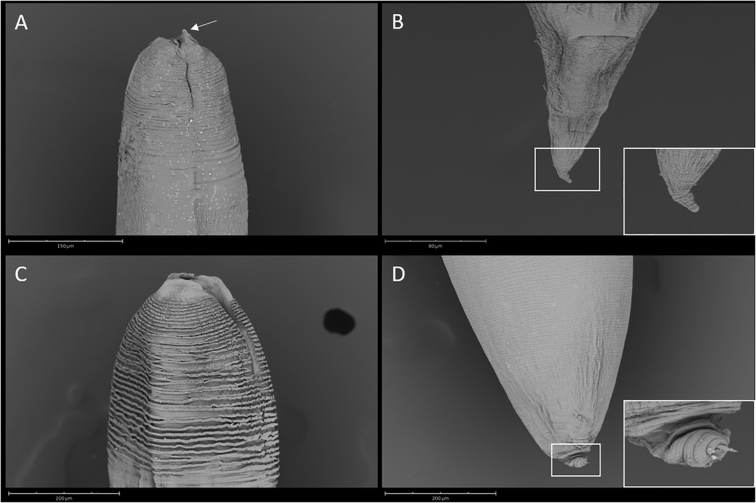
SEM micrographs of *Contracaecum* third-stage larvae from Sea of Galilee fish. (A) *C. quadripapillatum* anterior end showing a well-defined boring tooth (arrow). (B) *C. quadripapillatum* conical posterior end. (C) *C. multipapillatum* E anterior end. (D) *C. multipapillatum* E posterior end with detail of the pointed tip.

The *C. quadripapillatum* larvae were 3.8 ± 0.56 (3.4–4.2) cm long × 1 ± 0.016 (0.98–099) cm wide, with a finely striated cuticle along the whole body. Cuticular ridges were slightly more marked at the anterior end, interrupted by narrow lateral lines. Anterior end had three small lips and a well-defined boring tooth, 12.4 ± 0.56 (12–12.8) long (Fig. 1A). A subterminal excretory pore was very close to the oral opening. Narrow esophagus was present, 3286 ± 1087.4 (2516.9–4054.7) long; the intestinal cecum was 2569.1 ± 1198.1 (1722–3416.3) long, and the ventricular appendix was 773.8 ± 347.2 (528.3–1019.4) long, much shorter than the cecum; a conical tail was 86 ± 16.6 (74.2–97.7) long (Fig. 1B).

The two larvae of *C. multipapillatum* E were 4.5 ± 2.8 (4.3–4.7) cm long × 1.4 ± 0.29 (1.2–1.6) cm wide, with a stout body, tapering at both ends, and with cuticular ridges along the whole body, considerably more pronounced at the anterior end (Fig. 1C), interrupted by narrow lateral lines. The anterior end had three labial primordia and a short and faint boring tooth-like structure, 8.9 ± 1.99 (7.4–10.3) long. The subterminal excretory pore was very close to the oral opening; a narrow esophagus, 4301.1 ± 292.4 (4094.3–4508) long, ended in a small roundish ventriculus. Intestinal cecum, 3207.6 ± 208.9 (3059.9–3355.3) long, extended beyond a slightly visible nerve ring. The ventricular appendix, 970.1 ± 14.6 (959.7–980.4) long, was much shorter than the cecum. The tail, 59.5 ± 7.44 (54.2–64.7) long, was conical, ending in a pointed tip (Fig. 1D).

## 4 Discussion

Fishery products can harbor a variety of parasites, some of which may cause zoonotic diseases in humans, whereas others may disqualify the fish for marketing (Davidovich et al., 2022; Guardone et al., 2021). Fish catch infected with larval nematodes of the family Anisakidae, especially from a marine environment, are responsible for a rising number of human cases (Aibinu et al., 2019; Buchmann and Mehrdana, 2016). The occurrence of *C. osculatum s.l.* larvae has been reported in two human cases (Nagasawa, 2012); nevertheless, the zoonotic potential of the different *Contracaecum* species has yet to be proven (Shamsi and Butcher, 2011).

*C. quadripapillatum* (adults and larvae) was first described by Saad et al. (2018) after an experimental infection of American white pelican (*Pelecanus erythrorhynchos*) with L3 collected from North African catfish (*Clarias gariepinus*) (formerly *Clarias lazera*) that were captured from different areas of Lake Nasser (Egypt). Hamouda and Younis (2022) examined cichlids and silurids collected in the same area, reporting the presence of *C. quadripapillatum* only in the latter family. In the same year, Thabit and Abdallah (2022) reported a high prevalence of infection with *C. quadripapillatum* larvae in Nile perch (*Lates niloticus*) collected from the Nile River, Assiut Governorate (Egypt). In 2023, Caffara et al. published the first description of *C. quadripapillatum* adults collected from naturally infected great white pelican (*Pelecanus onocrotalus*) sampled elsewhere in Israel. In the present study, we confirm—for the first time—that wild native cyprinids from the Sea of Galilee, namely Jordan himri (*Carasobarbus canis*) and Jordan barbel (*Luciobarbus longiceps*) can be intermediate/paratenic hosts of *C. quadripapillatum* L3. Interestingly, in the literature, we found no reports of infection with *C. quadripapillatum* larvae in non-native edible cyprinids farmed in Israel, namely common carp (*Cyprinus carpio*), grass carp (*Ctenopharyngodon idella*), silver carp (*Hypophthalmichthys molitrix*) or black carp (*Mylopharyngodon piceus*). The appearance and morphometrics of *C. quadripapillatum* larvae analyzed in the present study, although based on a limited number of specimens, are in accordance with the description provided by Saad et al. (2018).

*C. multipapillatum* E was described for the first time, by morphological and molecular analyses, by Davidovich et al. (2022) in hybrid tilapia (*Oreochromis aureus* × *O. niloticus*) and red drum (*Sciaenops ocellatus*) farmed in Israel. This species has also been recorded in several wild fish species in African countries (Ethiopia, Egypt, and Kenya) by Otachi et al. (2014) and Younis et al. (2017). Those authors did not provide any species identification, which was confirmed by Davidovich et al. (2022) by comparison to the sequences available in GenBank. Thereafter, Caffara et al. (2023) confirmed, by morphological and molecular analysis, the presence of a *C. multipapillatum* E adult female from a great white pelican (*Pelecanus onocrotalus*) in Israel. In this study, we confirm the presence of *C. multipapillatum* E larvae in wild blue tilapia (*Oreochromis aureus*) collected from the Sea of Galilee.

The drastic reduction in the prevalence of *Contracaecum* larvae observed in our study compared to that reported by Paperna in 1964 in the same area and fish species raises several different scenarios. In general, heteroxenous parasites are strictly dependent on the occurrence of suitable intermediate and definitive hosts to complete their life cycle (Davidovich et al., 2022). This makes them sensitive bioindicators of environmental changes (Zhu et al., 2007; Dzikowski et al., 2003), because impacts on any of these hosts directly and indirectly affect the successful transmission of the parasite. Moreover, changes in host distribution, particularly in highly mobile or migratory species, can play a significant role in influencing the abundance and distribution of certain parasite species, as shown for *Contracaecum* (Mattiucci et al., 2020) and other anisakids (Cipriani et al., 2022).

Freshwater ecosystem biodiversity is well-known to be declining at an alarming and unexplainable rate due to anthropogenic activities (Brucet et al., 2013). Changes in lake morphometry (area and depth) are important natural factors that may influence fish communities (Brucet et al., 2013; Mehner et al., 2007). Many fish-eating birds, definitive hosts of *Contracaecum* spp., are migratory species, and their populations have steeply declined in the last few decades (Rosenberg et al., 2019). Schekler et al. (2022) reported that the distribution of migratory birds is primarily associated with artificial light at night, which is strongly correlated with high densities of migrants.

With reference to the Sea of Galilee, a recent paper by Shapiro et al. (2022) analyzed the changes in phyto-zooplankton composition in the lake due to modifications in their ecosystem related mainly to water-level variations. The composition of the phytoplankton was modified from a stable dinoflagellate system to an unstable cyanobacteria-abundant system, affecting the composition of the zooplankton and consequently, the fish populations and ecosystem stability. Dzikowski et al. (2003) analyzed limnological variation that was strictly connected to the life cycle of some digenetic trematodes sharing the snail *Bulinus truncatus* as first intermediate host. The authors observed a decline in the trematode parasites, possibly because the decline in lake water level eliminated the habitats which supported thriving populations of *B. truncatus*.

With respect to the present study, the dramatic reduction in the prevalence of *Contracaecum* spp. larvae from Lake Kinneret fishes could be related to changes in either the populations of intermediate aquatic hosts, as hypothesized in studies focused on other parasite species (Dzikowski et al., 2003), or the abundance and distribution of definitive host birds. In Israel, *Pelecanus onocrotalus* was found infected with adult stages of both *C. quadripapillatum* and *C. multipapillatum* E (Caffara et al., 2023), highlighting its role as definitive host of these *Contracaecum* species. *P. onocrotalus* migrate from the Danube Delta to wintering sites in Africa, stopping for several days in Israel to rest and feed; however, wetland deterioration has negatively affected long-distance migrating birds' ability to find suitable areas to stop over (Arad, 2021).

More accurate hypotheses on the observed changes in infection patterns of *Contracaecum* spp. in the considered area rely on the identification of possible intermediate hosts in the life cycle of *C. quadripapillatum* and *C. multipapillatum* E, and on the understanding of how different forms of anthropogenic impacts might affect their natural populations. To the best of our knowledge, no human cases of *Contraceacum* larvae have been described in Israel in the past and most freshwater fisheries are not used for uncooked dishes; nevertheless, public veterinarians and consumers of fishery products, especially of traditionally homemade (uncooked) products (e.g., salted or smoked fish), should be aware of the possible presence of *Contracaecum* spp. larvae in some fish species of the Sea of Galilee.

## Declaration of competing interest

The authors declare that they have no known competing financial interests or personal relationships that could have appeared to influence the work reported in this paper.
